# 吉非替尼可能通过*EGFR*启动子甲基化诱导肺腺癌细胞继发性耐药

**DOI:** 10.3779/j.issn.1009-3419.2015.04.04

**Published:** 2015-04-20

**Authors:** 起龙 王, 敏 李, 成平 胡

**Affiliations:** 410008 长沙，中南大学湘雅医院呼吸内科 Department of Respiratory Medicine, Xiangya Hospital of Central South University, Changsha 410008, China

**Keywords:** 肺肿瘤, 酪氨酸激酶抑制剂, 继发性耐药, 表皮生长因子受体, 甲基化, Lung neoplasms, Tyrosine kinase inhibitor, Secondary resistance, Epidemal growth factor receptor, Methylation

## Abstract

**背景与目的:**

在肺腺癌靶向治疗中的吉非替尼继发性耐药是临床遇到的重要问题。本研究旨在探讨吉非替尼是否可诱导肺腺癌PC9细胞发生继发性耐药，以及表皮生长因子受体（epidermal growth factor receptor, *EGFR*）启动子甲基化在耐药过程中的作用，拟为肺腺癌耐药提供新的治疗靶点。

**方法:**

体外培养肺腺癌吉非替尼敏感细胞株PC9，应用不同浓度吉非替尼进行干预。MTT法检测PC9细胞株对吉非替尼的敏感性。亚硫酸氢盐处理后测序法（bisulfite sequencing polymerase chain reaction, BSP）检测肺腺癌PC9细胞株*EGFR*启动子甲基化水平。使用5-氮杂-2’-脱氧胞苷（5-Aza-dc）对吉非替尼耐药细胞株PC9/GR细胞株进行干预。MTT法检测耐药株PC9/GR对吉非替尼敏感性的变化。

**结果:**

经过从0.01 μmol/L逐渐提升吉非替尼诱导浓度至3 μmol/L之后，MTT显示耐药细胞株PC9/GR细胞株半抑制浓度（half maximal inhibitory concentration, IC_50_）[（3.95±0.23）μmol/L]较之前敏感株PC9[(0.01±0.002) μmol/L]明显升高（*P* < 0.05），BSP显示发生异常甲基化的位点结果比较：PC9/GR甲基化水平明显升高（74% *vs* 59%, *P* < 0.05）。RT-PCR显示PC9/GR细胞株较PC9细胞株EGFR mRNA表达量升高（*P* < 0.05）。使用5-Aza-dc处理PC9/GR细胞后，PC9/GR细胞株IC_50_较对照组降低[（2.55±0.14）μmol/L *vs*（3.87±0.034）μmol/L, *P* < 0.05）]。

**结论:**

吉非替尼体外浓度递增诱导法可诱导PC9细胞株产生吉非替尼继发性耐药，成功构建PC9/GR耐药细胞株。PC9细胞EGFR启动子甲基化异常可能参与了吉非替尼继发性耐药机制。

## Abstract (en)

**Background and objective:**

Nowadays the secondary resistance of gefitinib in the treatment of lung adenocarcinoma is an outstanding problem. This research is to explore whether the gefitinib secondary resistance can be induced by gefitinib, to explore whether epidermal growth factor receptor (*EGFR*) promotor methylation correlate with the gefitinib-resistance in PC9/GR cell lines and to find a new therapeutic target to overcome the gefitinib secondary resistance in lung adenocarcinoma.

**Methods:**

*In vitro* cultivation of lung adenocarcinoma PC9 cell lines, apply gefitinib on lung adenocarcinoma PC9 cell lines, and improve drug concentration. MTT for test of gefitinib resistance index in PC9 cell and PC9/GR cell. Bisulfite sequencing polymerase chain reaction (BSP) and Reverse transcription-polymerase chain reaction (RT-PCR) for detection of *EGFR* promoter methylation status and mRNA expression. *In vitro* cultivation of lung adenocarcinoma PC9 cell lines, apply 1 μmol/L 5-Aza-dc on lung adenocarcinoma PC9/GR cell lines for 72 h. MTT method for test of gefitinib resistance index in PC9/GR cell.

**Results:**

After improving the gefitinib concentration, MTT results showed that half maximal inhibitory concentration (IC_50_) of PC9 cell lines increase from (0.01±0.002) μmol/L to (3.95±0.23) μmol/L (*P* < 0.05). BSP results showed that abnormal methylation sites compared the degree of methylation change: PC9: 59%; PC9/GR: 74% (*P* < 0.05). RT-PCR results showed in PC9/GR cell lines, EGFR mRNA expression quantity increased (*P* < 0.05). After applying 5-Aza-dc on PC9 cell lines, IC_50_ of PC9/GR decrease from (3.87±0.034) μmol/L to (2.55±0.14) μmol/L.

**Conclusion:**

The PC9 cell line which is induced by improving gefitinib concentration will be resistant to gefitinib, and the gefitinib-resistant cell line PC9/GR could be built. *EGFR* gene promoter methylation may be one of the mechanisms for the secondary resistance to gefitinib.

分子靶向治疗在肺癌的临床治疗中表现出了良好的应用前景。表皮生长因子受体酪氨酸激酶抑制剂（epidemal growth factor receptor-tyrosine kinase inhibitor, EGFR-TKI）研究^[1, 2]^结果显示，TKI治疗效果显著的患者存在基因突变，以19外显子缺失和21外显子的突变为主。然而随着EGFR-TKI治疗方案的临床推广，耐药问题逐渐出现，成为了新的研究热点。大多数*EGFR*突变阳性的非小细胞肺癌（non-small cell lung cancer, NSCLC）患者在初始治疗大约6个月-12个月后会出现耐药，从而导致TKI失效；而部分患者即使携带EGFR活化突变，仍对TKI不敏感。

临床发现的耐药主要包括原发性耐药和继发性耐药两大类，原发性耐药的主要机制为原发性耐药突变、次级药物暴露程度，细胞诱导凋亡障碍等。继发性耐药则主要包括了EGFR的二次突变、药物暴露程度，异常小分子激活EGFR通路、组织学类型转化等机制^[3-5]^。TKI继发性耐药已被认为有多重途径，以上几种机制均是在基因水平解释TKI继发性耐药。目前大量的证据表明基因二次突变是药物继发性耐药机制的重要组成部分。然而，基因组学在相对较快的耐药以及药物应答可逆性方面的解释仍然显得不那么充分。研究^[5, 6]^证实，部分耐药患者靶点基因未发现突变，也有一些患者旁路被激活。提示除基因组学机制外可能还存在其他继发性耐药机制。

表观遗传学是重要遗传学分支学科，主要研究表观遗传变异。表观遗传变异是指在基因的DNA序列没有改变，基因功能却发生可遗传的变化，并最终导致生物表型发生变化。甲基化则是表观遗传学研究中基因组DNA一种主要的表观遗传修饰形式，可调节基因组功能。DNA甲基化过程主要通过DNA甲基转移酶（DNA methyltransferase, DNMT）来催化。有研究表明：多种肺腺癌细胞株进行比较，对TKI敏感性高的细胞株EGFR启动子区域甲基化程度低，而敏感性低的细胞株对应甲基化程度高^[7]^。我们推断吉非替尼可能诱导PC9细胞对其产生继发性耐药，以及EGFR启动子甲基化可能在该过程中起到作用，以期为肺腺癌耐药提供新的治疗靶点。

## 1 材料与方法

### 1.1 材料

人肺腺癌PC9细胞株（对EGFR-TKI敏感）和耐药细胞株PC9/GR_0_（对EGFR-TKI耐药）购自广州肺癌研究所；液体RPMI-1640培养基、胎牛血清（FBS）购自美国Gibco公司；MTT购自南京凯基生物科技有限公司；5-Aza-dc（DNA甲基转移酶抑制剂）购自美国Sigma公司。总RNA抽提试剂Trizol购自美国Invitrogen公司；逆转录试剂盒购自日本东洋纺公司；EGFR探针引物和GAPDH探针引物购自上海生工生物工程有限公司；吉非替尼粉剂购自大连美仑生物技术有限公司，溶解于DMSO，制成实验相应浓度1, 000倍的溶液，分装保存于-20 ℃冰箱中备用，实验时用培养液稀释至目标浓度，使终溶液中DMSO体积分数低于0.1%。

### 1.2 细胞培养

将PC9细胞株培养于含有10%胎牛血清培养液中，接种于培养瓶，放于37 ℃培养箱中静置培养。次日换培养液，继续培养。每天观察细胞生长情况，每两天更换一次细胞培养液（含有10%胎牛血清的RPMI-1640），长满培养瓶后进行消化传代，待传代至3代后，80%贴壁的细胞用于实验。5-Aza-dc干预吉非替尼耐药细胞株PC9/GR取对数生长期的PC9/GR细胞进行试验，0.25%胰蛋白酶消化成细胞悬液，接种于细胞培养瓶中，分组如下：实验组：培养PC9/GR细胞，加入1 μmol/L 5-Aza-dc干预72 h，参考研究^[8]^；对照组：同期培养不加药的肺癌PC9/GR细胞，加入等容积的DMSO，作为5-Aza-dc干预前状态。待细胞贴壁后，对照组每24 h更换培养液一次，而实验组均每24 h更换含相应浓度5-Aza-dc的培养液一次。

### 1.3 MTT法检测PC9细胞半数抑制浓度

集对数期的细胞，调整悬浮液中细胞的浓度，每孔加入100 μL悬浮液，铺板使待测细胞调密度至8, 000/孔（边缘孔使用无菌PBS填充）。5%CO_2_、37 ℃孵育，至细胞单层贴壁（96孔板），加入浓度梯度药物吉非替尼（0 μmol/L, 0.01 μmol/L, 0.1 μmol/L, 1 μmol/L, 10 μmol/L, 100 μmol/L）。5%CO_2_、37 ℃条件下孵育48 h，使用倒置显微镜下观察。终止培养，然后小心吸去孔内培养液。这时每孔中快速加入150 μL DMSO，置于摇床上振荡10 min，充分溶解结晶物。在酶联免疫检测仪吸光度（optical delnsity, OD）550 nm处测量各孔的吸光值。

使用改良寇式法进行结果的计算，可得出细胞的半抑制浓度（half maximal inhibitory concentration, IC_50_）。具体公式为：lgIC_50_=Xm-I[P-(3-Pm-Pn)/4]。

### 1.4 耐药细胞株PC9/GR的制备

肺腺癌细胞株PC9（对EGFR-TKI敏感）购自广州呼吸病研究所。经过传代培养之后，取对数生长期的细胞进行实验，使用逐步提升浓度体外诱导法诱导其产生耐药。首先使用MTT法测定吉非替尼对敏感细胞PC9的IC_50_为（0.01±0.002）μmol/L，然后使用含有0.01 μmol/L吉非替尼的培养基进行培养48 h，之后更换不含有吉非替尼的培养基培养5 d-7 d，至细胞能够稳定生长并传代2次，此时再次测定IC_50_。然后使用含有0.02 μmol/L吉非替尼的培养基进行培养48 h，之后更换含有0.01 μmol/L吉非替尼的培养基培养5 d-7 d，至细胞能够稳定生长并传代2次，此时再次测定IC_50_。以此类推，逐渐提升药物浓度梯度为0.01 μmol/L、0.02 μmol/L、0.05 μmol/L、0.1 μmol/L、0.2 μmol/L、0.4 μmol/L、1 μmol/L、1.6 μmol/L、3 μmol/L。直至最后，细胞在含有3 μmol/L吉非替尼的培养基中可以稳定生长，最后检测此时处理后的吉非替尼的IC_50_值，将其命名为PC9/GR，并与所购买的PC9/GR_0_进行对比，以验证其是否达到吉非替尼耐药标准。

### 1.5 BSP法检测甲基化水平

取敏感株及耐药株细胞置于37 ℃、5%CO_2_培养箱中培养，72 h后回收，提取细胞DNA，使用亚硫酸盐进行甲基化修饰，之后再纯化亚硫酸盐修饰的DNA，然后以此为模板进行PCR扩增。甲基化特异性引物：上游5’-GTTATAGGGGTAGTGGGATATT-3’，下游5’-TAAACAAACTAACCR AACC TTA-3’。PCR反应体系包括10×PCR Buffer 2.5 μL，dNTP Mix 0.5 μL，PCR Primer（igf2 F/igf2 R）0.5 μL/0.5 μL，Template 1 μL，rTaq 0.5 μL，ddH_2_O 19.5 μL定容至25 μL。反应条件：94 ℃预变性5 min，94 ℃变性30 s、58 ℃退火30 s、72 ℃延伸30 s，共40个循环。将PCR产物与T载体进行连接，之后进行大肠杆菌的转化、重组菌的鉴定。使用BiQ Analyzer分析软件对DNA序列和测序序列进行比对。

### 1.6 逆转录PCR

取所培养的不同阶段细胞置于37 ℃、5%CO_2_培养箱中培养，72 h后回收。Trizol法提取细胞的总RNA，逆转录为cDNA，在实时荧光定量PCR仪扩增，GAPDH为内参。实时荧光定量PCR：反应体系20 μL体系中，SYBR qPCR Mix 10 μL，上下游引物Mix 1.2 μL（6 pmol），50X ROX reference 0.4 μL（ABI 7500RT-PCR仪器用0.4 μL），cDNA 4 μL，灭菌蒸馏水4.4 μL补齐体系。反应条件：95 ℃ 60 s预变性。95 ℃ 15 s，60 ℃ 60 s，进行40个循环。每组设置3个平行复孔，以△Ct值对结果进行分析。

### 1.7 统计学方法

实验进行中每组重复3次以上，使用SPSS 17.0统计软件进行数据分析。计量资料的检测结果均以均数±标准差（Mean±SD）表示，两组均数比较用*t*检验，率的比较采用卡方检验，*P* < 0.05为差异有统计学意义。

## 2 结果

### 2.1 *EGFR*基因启动子甲基化与PC9细胞吉非替尼耐药的相关性

#### 2.1.1 PC9及PC9/GR细胞形态学

倒置显微镜镜下见人肺癌细胞PC9（吉非替尼敏感细胞）（图 1A）与PC9/GR（吉非替尼耐药细胞）（图 1B）形态呈上皮细胞状，以贴壁方式生长，贴壁后呈长梭形，形态无明显区别。

**1:**
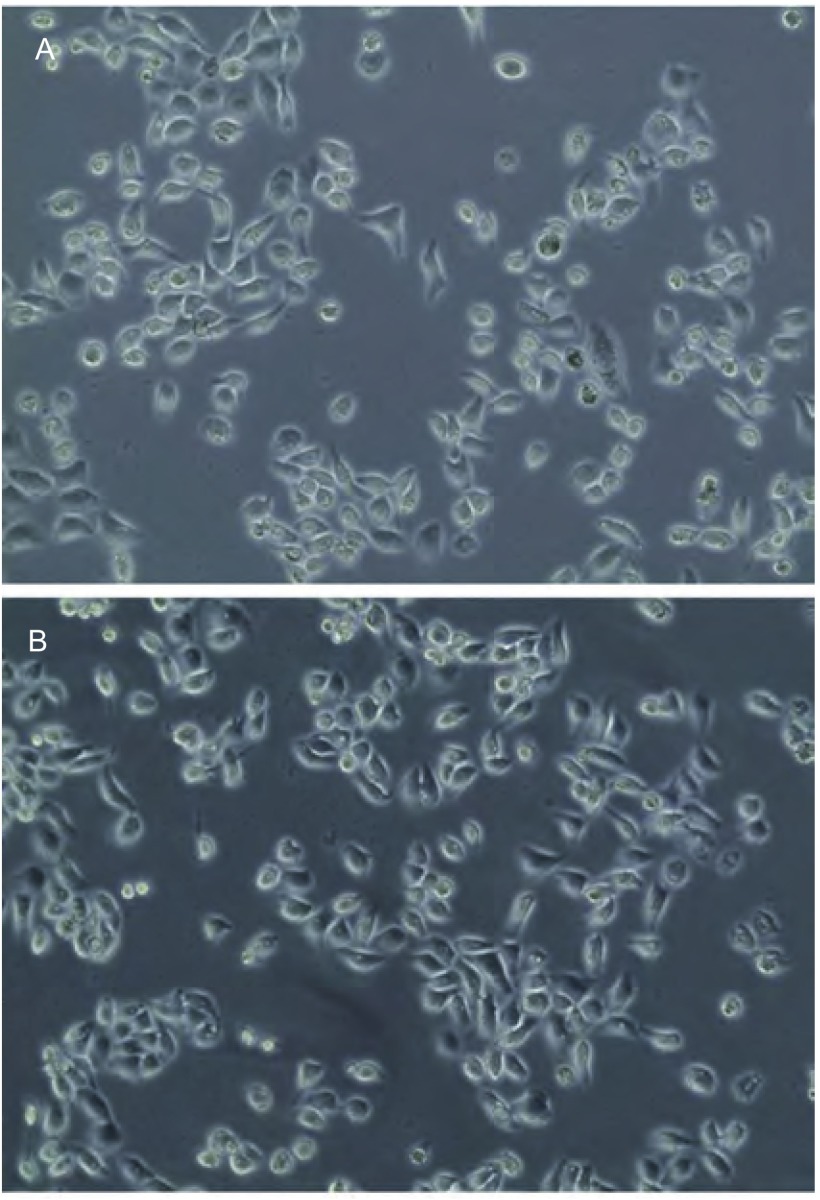
人肺腺癌细胞PC9（吉非替尼敏感细胞）（A）及人肺腺癌细胞PC9/GR（吉非替尼耐药细胞）（B） Human lung adenocarcinoma cell PC9 (gefitinib sensitive) (A) and human lung adenocarcinoma cell PC9/GR (gefitinib resistant) (B)

#### 2.1.2 PC9及PC9/GR细胞对吉非替尼的耐药性

##### 2.1.2.1 吉非替尼对PC9细胞增殖的影响

根据公式求出吉非替尼干预PC9细胞以及PC9/GR细胞48 h的半数抑制浓度IC_50_分别为（0.01±0.002）μmol/L、（3.95±0.23）μmol/L（表 1、表 2）。

**1:** 吉非替尼对PC9细胞增殖的影响 Influence of gefitinib to PC9's proliferation

	0 *μ*mol/L	0.01 *μ*mol/L	0.1 *μ*mol/L	1 *μ*mol/L	10 *μ*mol/L	100 *μ*mol/L
OD	0.76±0.10	0.26±0.03	0.18±0.02	0.16±0.03	0.14±0.02	0.08±0.01
IR (%)	0	72.68±0.05	84.33±0.05	86.78±0.05	89.71±0.05	98.8±0.05
IR: inhibitory rate; OD: optical density.						

**2:** 吉非替尼对PC9/GR细胞增殖的影响 Influence of gefitinib to PC9/GR's proliferation

	0 *μ*mol/L	0.01 *μ*mol/L	0.1 *μ*mol/L	1 *μ*mol/L	10 *μ*mol/L	100 *μ*mol/L
OD	0.67±0.13	0.64±0.11	0.54±0.07	0.45±0.06	0.41±0.08	0.13±0.02
IR (%)	0	4.53±0.05	21.4±0.05	35.47±0.05	42.27±0.05	88.34±0.05

##### 2.1.2.2 PC9/GR细胞对吉非替尼的耐药程度及诱导过程

PC9/GR对吉非替尼的耐药指数为吉非替尼对PC9/GR细胞的IC_50_除以吉非替尼对PC9细胞的IC_50_

经过吉非替尼逐步提升浓度体外诱导法干预肺癌敏感细胞株PC9，吉非替尼对于PC9细胞的半数抑制浓度随诱导浓度的上升而不断上升（图 2）。最终经过3 μmol/L吉非替尼干预后所得的PC9细胞根据上述公式算得耐药指数为395。由于其耐药指数大于300，且与所购买的耐药细胞株PC9/GR_0_的耐药指数（378）进行比较，耐药程度一致，成功建立模型，命名为PC9/GR细胞，确定为耐吉非替尼的肺癌细胞株，进行下一步的肺癌研究。

**2:**
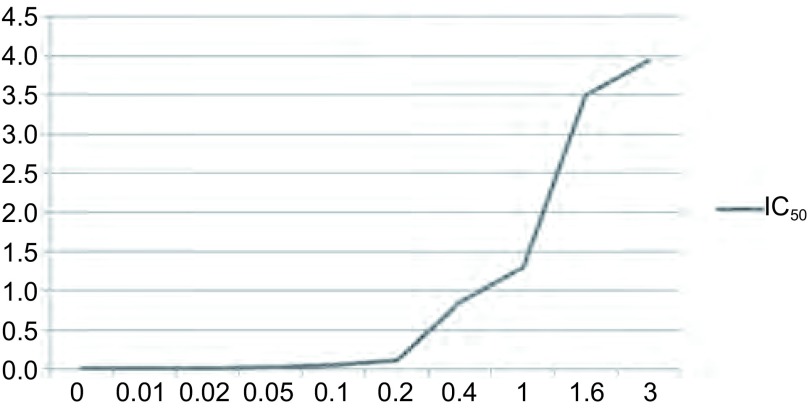
不同浓度（*μ*mol/L）吉非替尼诱导PC9细胞株耐药过程中IC_50_（*μ*mol/L） The half maximal inhibitory concentration (IC_50_) (*μ*mol/L) of PC9 cell in the process of gefitinib resistance

#### 2.1.3 PC9及PC9/GR细胞的*EGFR*基因启动子甲基化水平检测

研究检测了位于人类*EGFR*基因启动子区的甲基化水平，这一区域共检测出83个甲基化位点进行比较。每个样本经过亚硫酸氢盐处理后挑选10个左右克隆测序，结果所示，发生异常甲基化位点的甲基化水平变化PC9：59%；PC9/GR：74%，差异具有统计学意义（*P* < 0.05）（图 3）。

**3:**
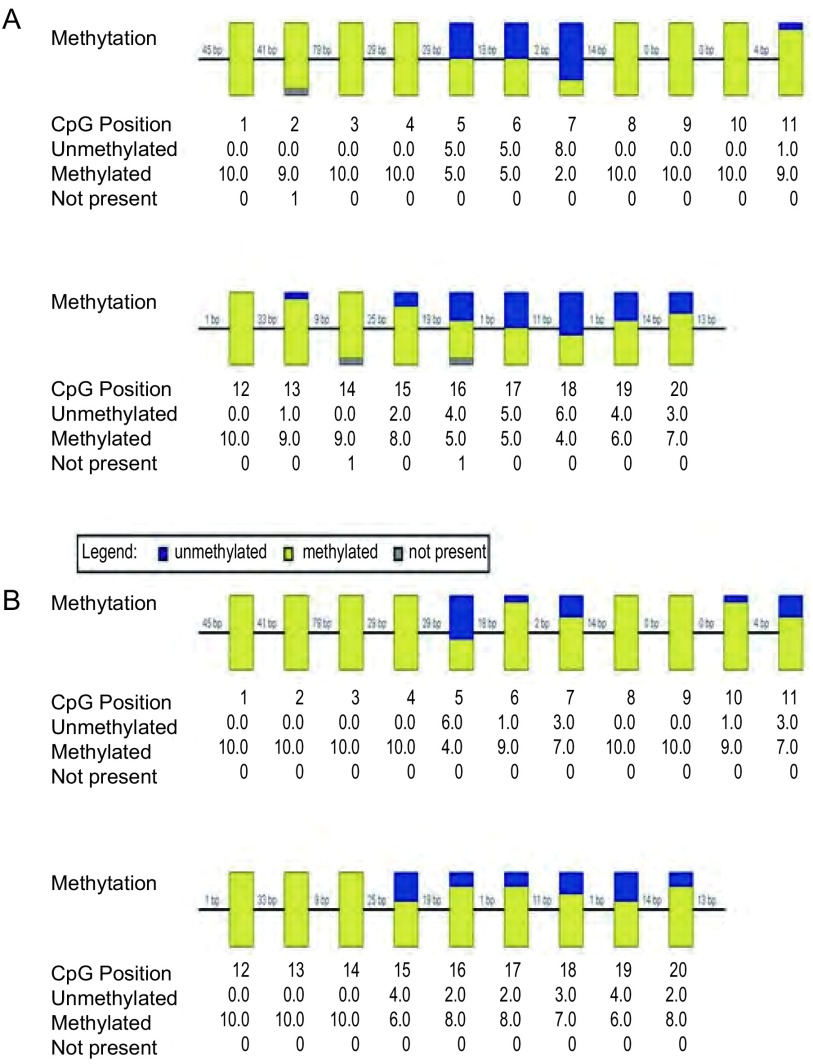
PC9细胞株*EGFR*启动子区域甲基化水平（A）及PC9/GR细胞株*EGFR*启动子区域甲基化水平（B） The methylation of *EGFR* promoter in PC9 cells (A) and the methylation of *EGFR* promoter in PC9/GR cells (B)

#### 2.1.4 PC9及PC9/GR细胞的*EGFR*基因表达

经过0.8 μmol/L及3 μmol/L吉非替尼处理得到的PC9/GR细胞株mRNA表达较未经过吉非替尼处理的敏感细胞株PC9明显增高，差异有统计学意义（*P* < 0.05）（图 4）。

**4:**
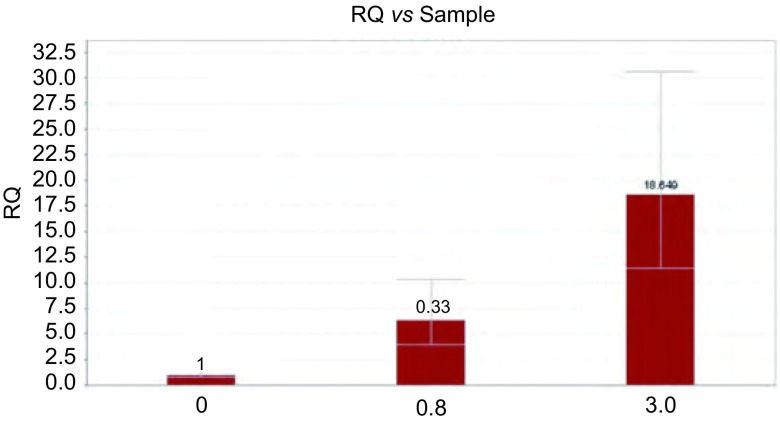
不同耐药程度PC9细胞EGFR mRNA水平 The epidermal growth factor receptor (EGFR) mRNA level of PC9 cell

### 2.2 5-Aza-dc干预PC9/GR细胞株TKI敏感性的逆转

#### 2.2.1 经5-Aza-dc处理后PC9/GR细胞形态学

在倒置显微镜镜下见人肺腺癌耐药细胞PC9/GR细胞（图 5A）形态呈上皮细胞状，贴壁方式生长，贴壁后呈长梭形，经5-Aza-dc处理后（图 5B）形态无明显区别。

**5:**
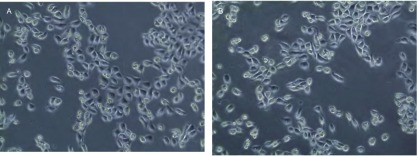
人肺腺癌耐药细胞PC9/GR细胞（A）和5-Aza-dc处理后人肺腺癌耐药细胞PC9/GR细胞（B） Human lung adenocarcinoma cell PC9/GR (A) and human lung adenocarcinoma cell PC9/GR after treated with 5-Aza-dc (B)

#### 2.2.2 经5-Aza-dc处理后PC9/GR细胞的耐药指数的变化

吉非替尼干预PC9细胞的IC_50_为（2.55±0.14）μmol/L（表 3）。对照组未经5-Aza-dc处理的PC9/GR细胞的IC_50_为（3.87±0.034）μmol/L（表 4）。两者相比较，差异有统计学意义（*P* < 0.05）。

**3:** 吉非替尼对干预组PC9/GR细胞增殖的影响 Influence of gefitinib to PC9/GR's proliferation in experiment group

	0 *μ*mol/L	0.01 *μ*mol/L	0.1 *μ*mol/L	1 *μ*mol/L	10 *μ*mol/L	100 *μ*mol/L
OD	0.69±0.09	0.65±0.11	0.56±0.05	0.48±0.06	0.44±0.08	0.14±0.02
IR (%)	0	4.82±0.03	22.13±0.05	37.51±0.03	46.23±0.04	93.78±0.06

**4:** 吉非替尼对对照组PC9/GR细胞增殖的影响 Influence of gefitinib to PC9/GR's proliferation in control group

	0 *μ*mol/L	0.01 *μ*mol/L	0.1 *μ*mol/L	1 *μ*mol/L	10 *μ*mol/L	100 *μ*mol/L
OD	0.66±0.15	0.65±0.12	0.55±0.04	0.46±0.03	0.42±0.05	0.12±0.01
IR (%)	0	4.52±0.03	21.39±0.06	35.52±0.04	42.19±0.04	88.22±0.09

## 3 讨论

本实验选用吉非替尼药物浓度递增的方法来进行耐药细胞模型的建立，最终结果显示其耐药性达到了耐药标准，且与购买的耐药细胞相比具有良好的一致。耐药肺癌细胞株PC9/GR的IC_50_较敏感株PC9的IC_50_明显升高，这种细胞模型优点在于更贴近临床患者产生耐药的过程，其造模时间约为4个月（第28代细胞），与临床出现耐药的时间大致吻合。

实验表明，PC9/GR细胞株甲基化水平与PC9细胞株相比，异常甲基化升高。TKI耐药的肺癌细胞其EGFR启动子区域甲基化水平较TKI敏感的肺癌细胞更高。在多种肿瘤已经证实，DNA启动子区域的甲基化状态和肿瘤耐药可能相关。Scartozzi等^[9]^进行52例结直肠癌的*EGFR*基因甲基化水平和EGFR单克隆抗体治疗敏感性之间关系的分析，结果显示，*EGFR*基因出现甲基化患者的疾病控制率和客观缓解率均显著低于*EGFR*基因未甲基化患者。Montero等^[10]^研究表明，存在有*EGFR*基因甲基化的乳腺癌细胞株CAMA1和MB453对吉非替尼敏感性低。EGFR表达量的变化可能是甲基化水平变化的一个影响结果。不同种类肺癌细胞株的EGFR mRNA表达水平和启动子甲基化水平存在差别，产生差异的分子机制尚不明确。另外，同一种细胞的EGFR启动子区域甲基化水平也可能有多种途径调控，甲基化也可能影响EGFR表达。本研究结果显示，耐药株细胞*EGFR*基因的mRNA表达量较敏感株高，与某些研究的不同种肺癌细胞株的结果相异，推测启动子甲基化不是影响EGFR mRNA表达的唯一原因。

实验结果提示经去甲基化药物5-Aza-dc处理之后，耐药细胞株PC9/GR对吉非替尼的敏感性有所回升，提示去甲基化药物5-Aza-dc作用于PC9/GR后，可以使其耐药性在一定程度上逆转，吉非替尼对PC9的抑制增殖作用加强。研究发现联合使用去甲基化药物5-Aza-dc可以增强原TKI不敏感肺腺癌细胞株H1650、H1299对吉非替尼的敏感性。同样类似的结果可以在其余肿瘤的治疗中发现。张晓伟等的研究提示，DNA甲基化和鼻咽癌细胞紫杉醇的耐药形成有关，使用5-aza-dc对鼻咽癌紫杉醇敏感细胞以及紫杉醇耐药细胞的增殖均有一定的抑制作用，而紫杉醇耐药细胞对其表现出更高的敏感性。经5-aza-dc处理之后的耐药细胞对紫杉醇药物的敏感性明显上升，从而可以部分程度逆转鼻咽癌对紫杉醇的化疗耐药^[11]^。5-Aza-dc是一种常用的去甲基化药物，其临床药物已经被美国食品药品监督管理局批准使用于血液系统恶性肿瘤治疗当中，在多种实体瘤的应用同时正在临床研究中，然而，由于其在各临床研究中所用剂量普遍较高，副作用较大，实体瘤的研究中并未得到预想的结果。然而，低剂量的应用方法被一些研究者认同。本试验采用低剂量干预^[8, 12]^，结果显示在一定程度上吉非替尼药物敏感性恢复。

总之，PC9细胞株继发性TKI耐药后，其EGFR启动子区域甲基化水平升高。使用去甲基化药物5-Aza-dc干预后，其对吉非替尼的敏感性在一定程度上恢复，其继发性耐药的出现可能与EGFR启动子区域甲基化水平升高有关。
